# Performance of the auxotrophic *Saccharomyces cerevisiae *BY4741 as host for the production of IL-1*β *in aerated fed-batch reactor: role of ACA supplementation, strain viability, and maintenance energy

**DOI:** 10.1186/1475-2859-8-70

**Published:** 2009-12-30

**Authors:** Lucia Paciello, Elisabetta de Alteriis, Cristina Mazzoni, Vanessa Palermo, Jesus Zueco, Palma Parascandola

**Affiliations:** 1 Dip to Ingegneria Chimica e Alimentare, Università di Salerno, Via Ponte Don Melillo, 84084 Fisciano, Salerno, Italy; 2 Dip to Biologia Strutturale e Funzionale, Università degli Studi di Napoli "Federico II", Via Cinthia, 80100 Napoli, Italy; 3 Dip to Biologia Cellulare e dello Sviluppo, Università di Roma "La Sapienza", P le Aldo Moro 5, 00185 Roma, Italy; 4 Unidad Microbiologia, Facultad de Farmacia, Universitat de Valencia, Avda Vicente Andrès Estelles 46100, Burjassot, Valencia, Spain

## Abstract

**Background:**

*Saccharomyces cerevisiae *BY4741 is an auxotrophic commonly used strain. In this work it has been used as host for the expression and secretion of human interleukin-1*β *(IL1*β*), using the cell wall protein Pir4 as fusion partner. To achieve high cell density and, consequently, high product yield, BY4741 [PIR4-IL1*β*] was cultured in an aerated fed-batch reactor, using a defined mineral medium supplemented with casamino acids as ACA (auxotrophy-complementing amino acid) source. Also the *S. cerevisiae *mutant BY4741 Δ*yca1 *[PIR4-IL1*β*], carrying the deletion of the *YCA1 *gene coding for a caspase-like protein involved in the apoptotic response, was cultured in aerated fed-batch reactor and compared to the parental strain, to test the effect of this mutation on strain robustness. Viability of the producer strains was examined during the runs and a mathematical model, which took into consideration the viable biomass present in the reactor and the glucose consumption for both growth and maintenance, was developed to describe and explain the time-course evolution of the process for both, the BY4741 parental and the BY4741 Δ*yca1 *mutant strain.

**Results:**

Our results show that the concentrations of ACA in the feeding solution, corresponding to those routinely used in the literature, are limiting for the growth of *S. cerevisiae *BY4741 [PIR4-IL1*β*] in fed-batch reactor. Even in the presence of a proper ACA supplementation, *S. cerevisiae *BY4741 [PIR4-IL1*β*] did not achieve a high cell density. The Δ*yca1 *deletion did not have a beneficial effect on the overall performance of the strain, but it had a clear effect on its viability, which was not impaired during fed-batch operations, as shown by the *k*_*d *_value (0.0045 h^-1^), negligible if compared to that of the parental strain (0.028 h^-1^). However, independently of their robustness, both the parental and the Δ*yca1 *mutant ceased to grow early during fed-batch runs, both strains using most of the available carbon source for maintenance, rather than for further proliferation. The mathematical model used evidenced that the energy demand for maintenance was even higher in the case of the Δ*yca1 *mutant, accounting for the growth arrest observed despite the fact that cell viability remained comparatively high.

**Conclusions:**

The paper points out the relevance of a proper ACA formulation for the outcome of a fed-batch reactor growth carried out with *S. cerevisiae *BY4741 [PIR4-IL1*β*] strain and shows the sensitivity of this commonly used auxotrophic strain to aerated fed-batch operations. A Δ*yca1 *disruption was able to reduce the loss of viability, but not to improve the overall performance of the process. A mathematical model has been developed that is able to describe the behaviour of both the parental and mutant producer strain during fed-batch runs, and evidence the role played by the energy demand for maintenance in the outcome of the process.

## Background

Among yeasts, *Saccharomyces cerevisiae *is one of the best established host systems for heterologous protein expression, due to the good knowledge of its physiology and genetics [1] and also the wide availability of fermentation technologies. However, other yeasts such as *Pichia pastoris *[2], *Kluyveromyces lactis *[3,4], and *Zygosaccharomyces bailii *[5-7], to name a few, have been successfully employed for heterologous protein production with, in general, better yields of secreted recombinant proteins, pointing the need for further refining of *S. cerevisiae *as a host system, specially in conditions resembling those of industrial production. Auxotrophic mutants of *S. cerevisiae *are used as host systems for heterologous protein production [8] because they ensure maintenance of plasmids with selectable markers [9].

In the present work, the auxotrophic *S. cerevisiae *BY4741 *(MATa, ura3Δ0, leu2Δ0, met15Δ 0, his 3Δ1)*, in which deletions were made in a genetic background isogenic to S288c (the *Saccharomyces cerevisiae *strain used in the systematic DNA sequencing project), has been used as host for human interleukin-1*β *(IL-1*β*) production in aerated fed-batch-reactor. IL-1*β *was expressed in *S. cerevisiae *BY4741 using the signal peptide and pro domain of the Pir4 cell wall protein [10], previously used to express and secrete into the medium a *Bacillus *sp.BP-7 xylanase A [11], the VP8* fragment of rotavirus capsid protein [12] and *Bacillus subtilis *lipase A [13].

The aerated fed-batch was chosen as cultivation system to exert, by means of sugar limitation, the metabolic control on *S. cerevisiae *BY4741, a glucose-sensitive yeast [14,15]. Indeed, sugar limitation avoids over-flow metabolism and favors oxidative metabolism with high yield of biomass and recombinant product [16].

Fed-batch operative conditions and the design of the fermentation strategy may affect the performance of the producer strain, in particular, agitation and aeration. With regard to this matter, only a small number of works in the literature state the importance of these process parameters during fermentation runs carried out with yeast cells [17,18].

In our work feeding strategy consisted in an initial phase of exponentially increasing feed followed by a constant feed. The oxygen transfer capacity of the bioreactor was enhanced through a cascade system, with the agitation speed automatically increasing with the increasing oxygen demand. A supplemented defined mineral medium was used for the cultivation, and the effect of the auxotrophy-complementing amino acid (ACA) concentration in the feeding solution was examined. The performance of the producer strain was evaluated in terms of total biomass and product; residual glucose and ethanol produced being monitored as well.

The unsatisfactory performance of BY4741 [PIR4-IL1*β*] in the aerated fed-batch reactor even with the proper ACA supplementation, led us to improve the host robustness. Aiming at this, a *S. cerevisiae *BY4741 mutant, which had been deleted for the *YCA1 *gene, was transformed with the expression vector containing the gene fusion *PIR4-IL1β *to obtain the producer strain *S. cerevisiae *BY4741 Δ*yca1 *[PIR4-IL1*β*]. *YCA1 *encodes a caspase-like protein (Yca1), involved in the initial triggering of the regulated process of apoptosis [19,20]. Yeast strains deleted for the *YCA1 *gene (Δ*yca1*) have shown a better survival after exposure to oxygen stress, salt stress or long-term culture [20].

The performance of *S. cerevisiae *BY4741 Δ*yca1 *[PIR4-IL1*β*] in the aerated fed-batch reactor was investigated as well, and its viability during the run was determined and compared to that of the parental producer strain.

An unstructured mathematical model was set-up to describe the time-course of the process for both the producer strains in fed-batch culture conditions. Modeling was developed starting from the bioreactor model represented in terms of a mass balance equation applied to the process variables of interest, *i.e*. biomass, residual limiting substrate (glucose) and product (IL-1*β*). Rates of cellular reactions of interest were described with suitable kinetic expressions which accounted for the loss of viability during the fed-batch runs, as well as the energy demand for both growth and maintenance.

## Results

### Growth of *Saccharomyces cerevisiae *BY4741 [PIR4-IL1*β*] and production of IL-1*β *in aerated fed-batch reactor

*S. cerevisiae *BY4741 [PIR4-IL1*β*] was cultivated in aerated fed-batch reactor using two feeding solutions which had a different ACA concentration, mentioned as FC and FAC in the text (see Materials and Methods for their composition). Feeding profile was designed to allow the producer strain to grow with a specific growth rate (*μ*) of 0.16 h^-1^.

**Figure 1:**
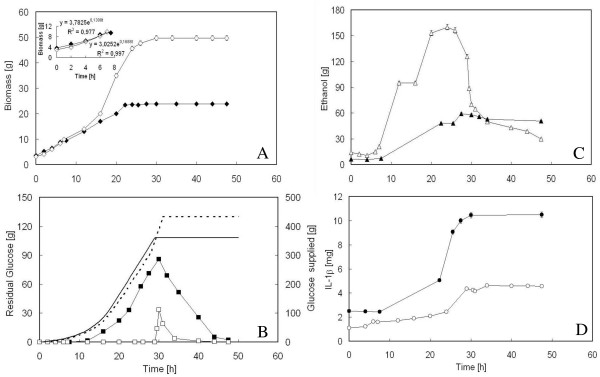
**Growth of *S. cerevisiae *BY4741 [PIR4-IL1*β*] in aerated fed-batch reactor, with either FC (full symbol) or FAC (empty symbol) solution in the feeding**. A: time-course of biomass; in the insert the interpolation of the experimental data regarding the biomass during the first 7 h of exponential feeding. B: time-course of residual glucose. Continuous and dashed lines are the profiles of glucose fed to the reactor in the case of FC and FAC solutions, respectively. During fed-batch runs, the feeding was maintained for 29 and 31 h for FC and FAC solutions, respectively, being marked by an initial exponentially increasing feeding of 19 h followed by a constant feeding of 10 and 12 h for FC and FAC solutions, respectively. C: time-course of ethanol produced. D: time-course of the product (IL-1*β*) secreted into the medium.

Figure 1 shows the time-course of biomass, residual glucose, ethanol and IL-1*β *during both the exponential and the constant feeding phase of fed-batch runs carried out with either FC or FAC solutions.

With FAC solution, the final biomass resulted twofold higher (50 g, corresponding to a cell density of 30 mg ml^-1^) than that obtained with FC (24 g, corresponding to a cell density of about 14 mg ml^-1^) (Figure 1A). However, even when FAC solution was used, growth progressively decreased during the run, and arrested after 29 h of feeding. With FC, growth arrest occurred even earlier (22 h).

Indeed, using FAC solution, a *μ *very near to that imposed in the feeding profile was maintained only during the first seven hours of feeding (Figure 1A, insert). During this period, the biomass yield (0.5 g biomass g^-1 ^glucose) was indicative of a fully respiratory metabolism. Contrarily, using FC solution, a lower *μ *of 0.13 h^-1 ^was exhibited in the same time interval (Figure 1A, insert), and the biomass yield was 0.35 g biomass g^-1 ^glucose. The values of biomass yield on glucose reported above were correctly determined, since the ethanol produced during the batch phase on the initial glucose charge and present in the reactor at the beginning of the fed-batch phase (Figure 1C), was not metabolized concomitantly with glucose.

If the fed-batch run was carried out using a feeding solution without casamino acids, but containing each individual auxotrophy-complementing amino acid in a concentration equal to that present in FAC solution, the total biomass achieved was 37 g (data not shown). This result points out the importance of using a complex nitrogen source in the feeding to improve the performance of the producer strain.

As regards glucose, the fed-batch culture carried out with FC solution was characterized by an earlier significant accumulation of the carbon source in the medium, whereas with FAC solution, residual glucose was null until 29 h and then accumulated (Figure 1B). Apparently, glucose supplied to the reactor was better consumed in the presence of a higher ACA concentration in the feeding. Once accumulated, glucose was rapidly consumed after the stop of the feed (Figure 1B), without any net increase in biomass (Figure 1A).

Glucose accumulation during the run was coupled with ethanol production (Figure 1C), indicating that the metabolism of the producer strain unavoidably shifted towards fermentation, even in the presence of the higher ACA concentration in the feed.

Differently from biomass, IL-1*β *did not seem to increase with the increase in ACA concentration, since a higher amount of product was achieved when FC was used (Figure 1D). It seems reasonable to suppose that the significant concentration of ethanol produced during the run with FAC solution (up to 20% v/v, Figure 1D), would have a denaturing effect on IL-1*β*, thus lowering the amount of protein detectable by immublot. Proteolytic activity of extracellular proteases secreted into the medium was not considered for a possible degradation of IL-1*β*, because extracellular protease activity at pH 5.00, which was the pH value during the runs, is relatively low [21].

### Growth of *Saccharomyces cerevisiae *BY4741 *Δyca1 *[PIR4-IL1*β*] and production of IL-1*β *in aerated fed-batch reactor

To improve the robustness of the producer strain, *S. cerevisiae *BY4741 Δ*yca1 *mutant was transformed with the expression vector pIA1 [11], carrying the gene fusion *PIR4-IL1β*. The transformed BY4741 Δ*yca1 *[PIR4-IL1*β*] was cultured, as the parental strain, in aerated fed-batch reactor using either FC or FAC as feeding solutions. The exponential feeding profile was built up to allow the mutant to grow with a specific growth rate (*μ*) of 0.16 h^-1^.

**Figure 2:**
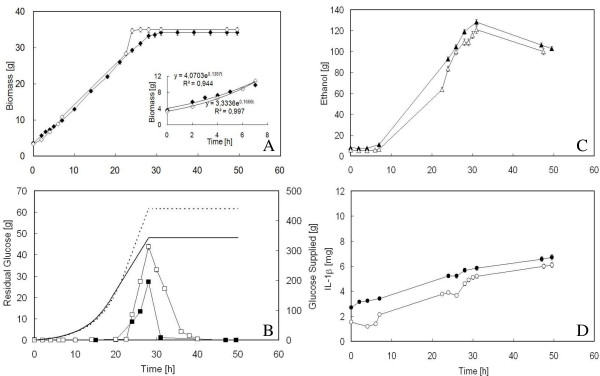
**Growth of *S. cerevisiae *BY4741 *Δyca1 *[PIR4-IL1*β*] in aerated fed-batch reactor, with either FC (full symbol) or FAC (empty symbol) solution in the feeding**. 2A: time-course of biomass; in the insert the interpolation of the experimental data during the first 7 h of exponential feeding. 2B: time-course of residual glucose. Continuous and dashed lines represent the profiles of glucose fed to the reactor in the case of FC and FAC solutions, respectively. During fed-batch runs, the feeding was maintained for 28 h, being marked by an initial exponentially increasing feeding of 19 and 22 h followed by a constant feeding of 9 and 6 h for FC and FAC solutions, respectively. 2C: time-course of ethanol produced. 2D: time course of the product (IL-1*β*) secreted into the medium.

Figure 2 shows the time-course of biomass, residual glucose, ethanol and IL-1*β *during both the exponential and constant feeding phase of the fed-batch runs carried out with BY4741 Δ*yca1 *[PIR4-IL1*β*].

Contrary to all expectations, the value of the final biomass achieved (Figure 2A) was the same regardless which solution was used and lower than that obtained with the parental strain grown with FAC (Figure 1A). Further, all the variables considered changed with time by moving almost in parallel, as if they were not affected by the ACA concentration employed (Figures 2A, B, C, D).

As far as biomass is concerned (Figure 2A), the only differences encountered between the two feeding solutions, regarded the ability to grow with the same *μ *value used to draw the exponentially increasing feeding profile (Figure 2A, insert) and the time of growth arrest. In fact, with FAC solution, the *μ *value kept very near to that imposed (Figure 2A, insert) at least in the first seven hours of feeding, but the growth arrest occurred more precociously with respect to FC. Apparently, a higher ACA concentration in the feed favoured the growth but only up to the achievement of a total biomass equal to FC.

Also in the case of the Δ*yca1 *mutant, glucose accumulation during the run (Figure 2B) was coupled with ethanol production (Figure 2C), indicating the shift towards fermentative metabolism. When the feeding was stopped, both glucose and ethanol were consumed, without any net increase in biomass (Figure 2A).

As regards IL-1*β *(Figure 2D), the denaturing effect of ethanol produced during the run and already observed in the case of BY4741 [PIR4-IL1*β*], seemed to persist, being the maximum values of the product close to those obtained with the parental strain when FAC solution was employed (Figure 1D).

**Figure 3:**
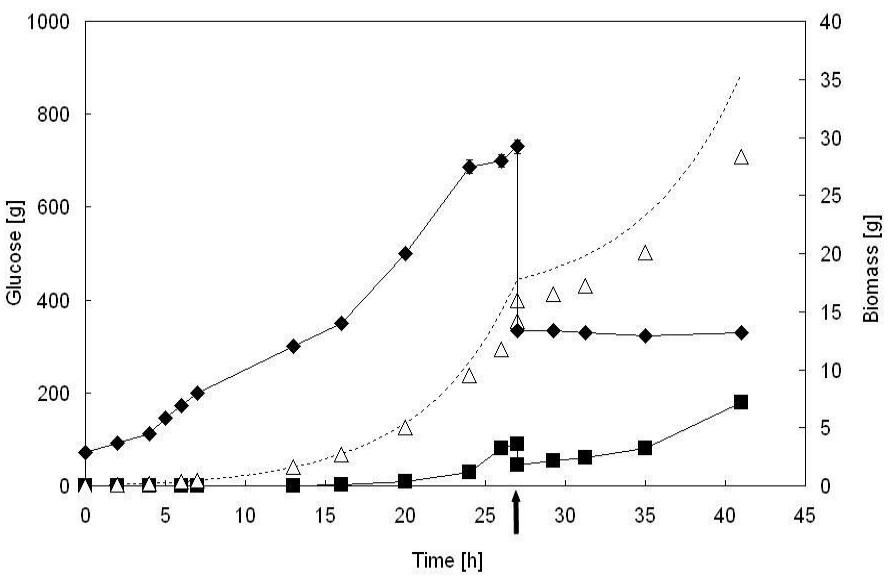
**Emptying and re-filling experiment with *S. cerevisiae *BY4741 *Δyca1 *[PIR4-IL1*β*]**. The figure shows the glucose fed to the reactor (dashed line), the residual (full square) and the consumed glucose (empty triangle), and the biomass (full rhomb) during fed-batch runs carried out with FAC solution. After 27 h run (indicated by an arrow), the vessel was half-emptied and a new exponentially increasing feed was started; it was designed as if the amount of biomass in the reactor remained unchanged.

To evaluate if a higher glucose concentration was needed to allow the *Δyca1 *mutant to continue to grow, an emptying-refilling experiment was performed. To this purpose, the reactor, at the end of a fed-batch run carried out with BY4741 Δ*yca1 *[PIR4-IL1*β*] and FAC solution, was half-emptied and fed again to start a second fed-batch phase (Figure 3). The second exponentially increasing feeding profile was built up to supply the reactor with an amount of glucose calculated as if the biomass remained unvaried after the culture vessel was emptied. Using this strategy, the glucose fed to the reactor would be high enough to ensure the saturation of transport systems for glucose and, consequently, yeast growth. Figure 3 shows the result of the experiment: glucose was consumed without any net change in biomass, suggesting that it might be used to satisfy an increasing energy demand for maintenance rather than to promote biomass production. A similar behaviour was observed when the parental BY4741 [PIR4-IL1*β*] strain was subjected to the same emptying-refilling experiment (data not shown).

### Viability of *Saccharomyces cerevisiae *BY4741 [PIR4-IL1*β*] and *S. cerevisiae *BY4741 *Δyca1 *[PIR4-IL1*β*] during fed-batch runs

**Figure 4:**
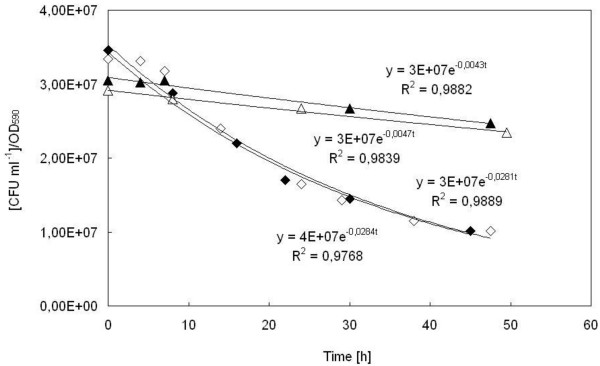
**Viability loss in aerated fed-batch reactor**. Viability of *S. cerevisiae *BY4741 [PIR4-IL1*β*] (full and empty rhombs) and *Δyca1 *mutant (full and empty triangles) was monitored during fermentation runs in aerated fed-batch reactor, and *k*_*d *_values were determined from the slope of the curves (CFU ml^-1^)/OD_590 _*vs*. time, when either FC (full symbols) or FAC (empty symbols) were used as feeding solutions.

The viability of the two strains, the parental *S. cerevisiae *BY4741 [PIR4-IL1*β*] and the mutant *S. cerevisiae *BY4741 Δ*yca1 *[PIR4-IL1*β*], was evaluated during both the exponential and constant feeding phase of the fed-batch runs carried out using either FC or FAC as feeding solutions. To this purpose, aliquots of broth culture of both the parental and the Δ*yca1 *mutant strain, were harvested at defined time intervals during the runs and, after suitable dilution, spread on selective agar plates for viable cell count. The amount of viable cells, expressed as CFU ml^-1^/OD_590_, was plotted *vs*. time (Figure 4).

The figure shows that the amount of viable cells diminished during the runs according to a first order kinetics, so that it was possible to calculate a specific death rate constant (*k*_*d*_). In the case of the parental strain, *k*_*d *_was 0.028 ± 0.0001 h^-1^, whereas a much lower value was calculated (*k*_*d *_= 0.0045 ± 0.0002) in the case of the Δ*yca1 *mutant, indicating that its viability was not so affected during the runs. The *k*_*d *_values obtained resulted independent of the ACA concentration employed in the feed for both the strains examined.

**Figure 5:**
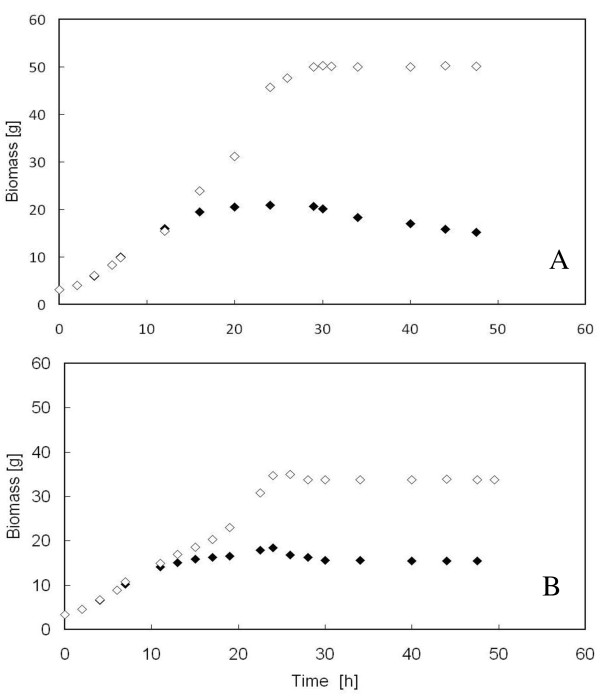
**Time-course of total and viable biomass of *S. cerevisiae *BY4741 [PIR4-IL1*β*] (A) and *S. cerevisiae *BY4741 *Δyca1 *[PIR4-IL1*β*] (B) in aerated fed-batch reactor**. Total (empty rhomb) and viable (full rhomb) biomass were determined spectrophotometrically and by viable count, respectively, during fed-batch runs carried out using FAC solution.

The viable cell count was also used to keep track of the viable biomass present in the reactor. Figure 5 shows that, along the first seven hours of run with FAC solution, the data regarding viable and total biomass of both the parental (Figure 5A) and the mutant (Figure 5B) strain overlapped, since all the biomass in the reactor kept viable; from that time onwards, the data pertaining to viable biomass deviated from those of total biomass, indicating that cell viability progressively reduced. In the case of the parental strain (Figure 5B), the gap between total and viable biomass was larger due to the higher *k*_*d *_value (Figure 4).

### Bioprocess modeling: a tool to investigate the behaviour of *S. cerevisiae *BY4741 [PIR4-IL1*β*] and *S. cerevisiae *BY4741 *Δyca1 *mutant in the aerated fed-batch reactor

To describe yeast behaviour in the aerated fed-batch reactor and provide a tool for optimization and control of the process, a mathematical model was developed, able to trace the time-course of the variables of interest (viable biomass, residual glucose and IL-1*β*) during the fed-batch runs carried out with both the parental and mutant BY4741 [PIR4-IL1*β*] strain. In the model, only the viable biomass was considered, being the only one able to reproduce, consume the glucose and synthesize the product.

The simulation curves concerning the viable biomass density, as well as the residual glucose and product concentrations were obtained starting from mass balance equations on the variables of interest and solving numerically the differential equations from given initial conditions (Table 1).

**Table 1:** Modeling of fed-batch reactor for both the parental *S. cerevisiae *BY4741 [PIR4-IL1*β*] and the mutant *S. cerevisiae *BY4741 Δ*yca1 *[PIR4-IL1*β*]: balance equations and kinetic expressions for the variables of interest.

Variable	Balance Equation	Kinetic Expression	
**Biomass**		*μ*_1 _= constant	0 ≤ *t *<*t*_1_
		*μ*_2 _= *μ*_1 _exp [-*δ *(*t*-*t*_1_)]	*t *≥ *t*_1_
		k_d _= constant	∀ t
**Glucose**		= constant	0 ≤ *t *<*t*_1_
		= exp [-*λ *(*t*-*t*_1_)]	*t *≥ *t*_1_
		= constant 0	≤ *t *<*t*_1_
		= a(*t*-*t*_1_)^2 ^+ *β *(*t*-*t*_1_) + *γ*	*t*_1 _≤ *t *<*t*_2_
		q_m3 _= constant	t ≥ *t*_2_
**Product** **(IL-1*β*)**		*Y*_*P*/*X *_= constant	∀ t

**Figure 6:**
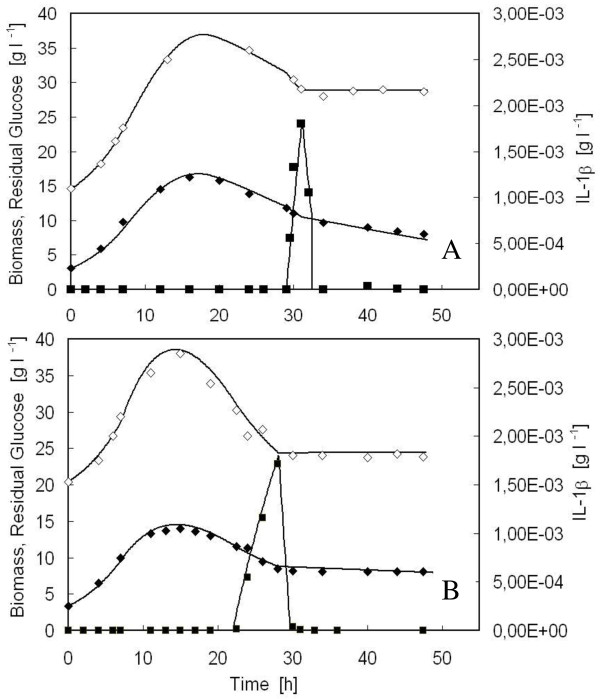
**Modeling of fed-batch reactor: cell density, residual glucose and product concentrations of *S. cerevisiae *BY4741 [PIR4-IL1*β*] (A) and *S. cerevisiae *BY4741 *Δyca1 *[PIR4-IL1*β*] (B)**. Simulation curves (continuous lines) and experimental data refer to viable biomass (empty rhomb), residual glucose (full square), and product concentration (full rhomb). **(A)**, initial values (t = 0): X_V0 _= 3,08 g l^-1^, V_0 _= 1 l, F_0 _= 1.98*10^-3 ^l h^-1^, G_0 _= 0, G_R _= 500 g l^-1^, P_0 _= 1.09*10^-3 ^g l^-1^. **(B)**, initial values (t = 0): X_V0 _= 3,37 g l^-1^, V_0 _= 1 l, F_0 _= 2.21*10^-3 ^l h^-1^, G_0 _= 0, G_R _= 500 g l^-1^, P_0 _= 1.53*10^-3 ^g l^-1^.

Figures 6 shows the good agreement existing between the simulation curves of the variables generated using the parameter values of Table 2 and the experimental data obtained with either the parental (Figure 6A) and the Δ*yca1*mutant (Figure 6B) strain. It is worth observing that the profiles of viable cell density for the two strains are comparable, accounting for a similar product formation. In both cases, the cell density achieves a maximum at around 15 h of fermentation run, then it significantly diminishes.

**Table 2:** Parameters values for modelling fed-batch reactor carried out with the parental *S. cerevisiae *BY4741 [PIR4-IL1*β*] and the mutant *S. cerevisiae *BY4741 Δ*yca1 *[PIR4-IL1*β*].

Parameter	Parental strain	*Δyca1 *mutant	Determination
*t*_1_	7 h	7 h	Experimental
*t*_2_	19 h	22 h	Experimental
*μ*_1_	0.18 h^-1^	0.16 h^-1^	Experimental
*k*_*d*_	0.026 h^-1^	0.005 h^-1^	Experimental
*δ*	0.126 h^-1^	0.131 h^-1^	by fitting
*λ*	0.139 h^-1^	0.146 h^-1^	by fitting
*α*	0.004 h^-3^	0.079 h^-3^	by fitting
_*β*_	0.025 h^-2^	0.006 h^-2^	by fitting
*γ*	0.039 h^-1^	0.081 h^-1^	by fitting
	0.313 h^-1^	0.307 h^-1^	Experimental
	0.007 h^-1^	0.013 h^-1^	by fitting
	1.03 h^-1^	1.76 h^-1^	by fitting
*Y*_*P*/*X*_	0.0001	0.0001	Experimental

**Figure 7:**
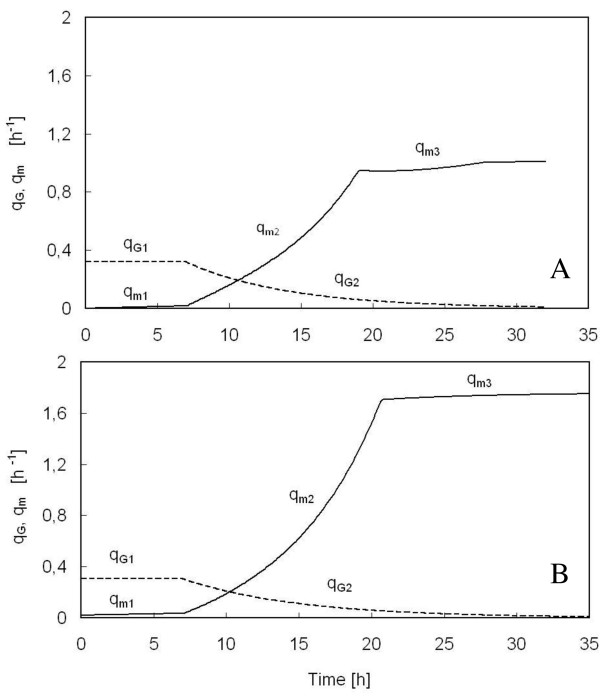
**Time-course of specific glucose consumption rate for both growth (q_G_) and maintenance (q_m_) of *S. cerevisiae *BY4741 [PIR4-IL1*β*] (A) and *S. cerevisiae *BY4741 *Δyca1 *[PIR4-IL1*β*] (B) in aerated fed-batch reactor**. q_G _(dashed line) and q_m _(continuous line). The curves have been obtained by the model reported in Figure 6.

As regards residual glucose, before it accumulates in the medium, its concentration is approximately null for both the parental and the mutant strain (Figure 6A and Figure 6B), even though the specific growth rate progressively decreased after seven hours of feeding (Figure 1A and 2A). This suggested that a large portion of the glucose supplied to the reactor was not converted into biomass, but diverted to satisfy an increasing energy demand for maintenance. For this reason, in the mathematical model both were considered, the specific glucose consumption rate to obtain energy and precursors to be channelled into anabolic pathways (q_G_) and that needed to satisfy an increasing energy demand for maintenance (q_m_) (Table 1). In Figure 7, the simulation curves regarding the time-course of the two specific glucose consumption rates, q_G _and q_m_, built up with the parameters of Table 2 for the parental (Figure 7A) and mutant strain (Figure 7B), are reported. q_G _is constant during the first seven hours of run (q_G1_), then decreases exponentially with time (q_G2_), similarly to what happens to the specific growth rate (Table 1), whereas the specific glucose consumption for maintenance (q_m1_), negligible in the first seven hours, increases according to a parabolic law (q_m2_), until the feeding is shifted into a constant feed. At that time, glucose fed to the reactor is insufficient even for maintenance, and q_m _turns to be constant (q_m3_). It is worth noticing that q_m2 _of the *Δyca1*mutant increases much more significantly than that of the parental strain.

## Discussion

*Saccharomyces cerevisiae *BY4741 is an auxotrophic yeast strain, commonly used for genetic experiments and molecular biology manipulations by the yeast research community http://web.uni-frankfurt.de/fb15/mikro/euroscarf/. In our group, fusion with the cell wall protein Pir4 [10] has been used for the expression of *Bacillus *sp. BP-7 xylanase A [11], *Bacillus subtilis *lipase A [13] and the VP8* fragment of the rotavirus spike protein [12], all of them using this particular yeast strain as host.

In the present paper, in order to achieve high cell density and, consequently, high product concentration in the medium, BY4741 [PIR4-IL1*β*] was cultured in an aerated fed-batch reactor, employing a defined mineral medium containing vitamins, trace elements and supplemented with casamino acids as amino acid source. The auxotrophy-complementing amino acid (ACA) concentration in the feeding was initially evaluated following the recommendations on the nutritional requirements reported in the literature [22,23]. It was shown that the concentration of ACA corresponding to the FC solution is limiting for the growth of *S. cerevisiae *BY4741 [PIR4-IL1*β*], the accumulation of glucose was precocious and massive, and growth early arrested during the run. To prevent this, the ACA concentration was increased (FAC solution), taking into account the value of biomass yield for the given amino acid in aerobic growth conditions and considering an excess factor of ca. 25%, to avoid "hidden" nutrient limitation. In fact, an excess factor of 25% is recommended, since auxotrophic strains may display a requirement of supplements even higher than that estimated from biomass yield and biomass composition, due to metabolic interferences and/or environmental conditions [24].

Although a higher ACA concentration promoted yeast growth, the strain was unable to display a fully oxidative metabolism for long and the ethanol produced during the run had a denaturing effect on IL-1*β*. *S. cerevisiae *BY4741 [PIR4-IL1*β*] did not achieve a high cell density, and its performance in the aerated fed-batch reactor was still considered unsatisfactory.

It is known that both metabolic and environmental stresses can affect the performance of a host system used in heterologous protein production [25]: the first one mainly due to the abuse of the cellular systems for the production of a foreign protein (the so-called "metabolic burden"), and the second imposed by the cultivation conditions largely depending on the fermentation strategy. In the present work, the possibility of a metabolic stress regarding *S. cerevisiae *BY4741 [PIR4-IL1*β*] can be excluded because the untransformed *S. cerevisiae *BY4741 exhibited a *μ*_max _value (0.40 h^-1^) and a performance in fed-batch reactor with FAC solution (49 g final biomass) comparable at all to those of the transformed strain. Apparently, the expression of interleukin-1*β *(MW 17000) did not determine a significant diversion of biosynthetic precursors normally available for biomass production.

Conversely, as regards the environmental stress, it has to be considered that fed-batch operations are generally carried out under conditions of vigorous and continuous agitation and aeration, so that shear and oxygen stress are almost unavoidable [25].

In this work, the weak performance of the auxotrophic *S. cerevisiae *BY4741 [PIR4-IL1*β*] was assumed to depend on an undefined cell damage caused by the stressful environmental conditions arising from the prolonged aeration. To test this hypothesis and aiming at improving the performance of *S. cerevisiae *BY4741, the mutant BY4741 Δ*yca1*, deleted for the *YCA1 *gene coding a caspase-like protein, was transformed with the expression vector containing the gene fusion *PIR4-IL1β *and cultured in the aerated fed-batch reactor. The results obtained have shown that the performance of the mutant BY4741 Δ*yca1 *[PIR4-IL1*β*] was not enhanced and resulted practically unaffected by an increased ACA supplementation, whereas its viability clearly improved with respect to that of the parental strain. This indirectly supported the hypothesis of stressful conditions (probably oxidative stress) occurring during the process and able to affect the parental strain viability. In fact, under oxygen stress, the parental cells would induce the expression of *YCA1 *gene to produce a protein, caspase, involved in the initial triggering of the regulated process of PCD [19,20].

Independently of their different robustness, both the parental and Δ*yca1 *mutant ceased to grow early in the run, because they would use most of the available carbon source to satisfy an increasing energy demand for maintenance in order to preserve all fundamental reactions [26] and not to promote biomass production. It has been reported that during conditions of external stress, maintenance energy requirements may increase considerably in *Saccharomyces cerevisiae *cells [27].

The mathematical model was instrumental in highlighting the role played by the energy demand for maintenance in the growth arrest. In fact, by modeling the bioprocess, it was evidenced that the energy demand for maintenance increased during cultivation and even more significantly in the case of the Δ*yca1 *mutant, accounting for the lower value of the final biomass and the earlier growth arrest with respect to the parental strain, even in the presence of an optimal ACA supplementation. Apparently, deletion of caspase gene visibly improved the strain robustness at the expense of a highly increased energy demand for maintenance.

As things stand, it would be more convenient, in the process conditions described, to stop the production when the energy request for maintenance begins to be burdening, that is when the specific growth rate of the producer strain significantly decreases.

In the light of the results presented, the auxotrophic commonly used *S. cerevisiae *BY4741 strain deserves attention when it is employed under conditions that resemble those of a production process. It has been reported that auxotrophic host systems may have physiological alterations and sensitivities which are not generally recognized [28]. Apparently, mutations by deletion in auxotrophic strains may give 'collateral' effects on one or more adjacent genes that can determine unexpected behaviour [29]. In our experience, in fact, the prototrophic *S. cerevisiae *S288c employed in the same operative conditions as BY4741 (aerated fed-batch reactor and defined mineral medium), showed a typical performance, achieving a cell density of 80 mg ml^-1^.

## Conclusions

The present work analyses the performance of the auxotrophic *S. cerevisiae *BY4741 as producer of human IL-1*β*. The importance of a correctly formulated auxotrophy-complementing amino acid solution for maximum efficiency of the process is highlighted in the work, as is the fact that cell viability is drastically reduced during the fed-batch run. The use of a BY4741 Δ*yca1 *mutant strain did reduce the extent of cell viability loss, but the overall performance of the process did not improve. The mathematical model suitably developed was able to describe the behaviour of *S. cerevisiae *BY4741 strain in fed-batch reactor, evidencing the role played by the energy demand for maintenance in the operative conditions adopted.

## Methods

### Strains and culture media

*Saccharomyces cerevisiae *cells, BY4741 strain *(MATa, ura3Δ0, leu2Δ0, met15Δ0, his 3Δ1) *(EUROSCARF collection, Heidelberg, Germany), transformed with the expression vector pIA1 [11] derived from the multycopy plasmid YEplac 195, containing *URA3 *as selectable marker and the human interleukin-1*β *(*IL-1β*) gene functionally fused with a portion of *PIR4 *ORF coding for a disulfide bound cell wall protein [10], was employed in this work and indicated as *S. cerevisiae *BY4741 [PIR4-IL1*β*]. The same expression vector pIA1 was employed to transform the deletion mutant *S. cerevisiae *BY4741 Δ*yca1 *(*MATα, ura3Δ0, leu2Δ0, met15Δ0, his3Δ1, yor197w::kanMX4) *[20]. Yeast transformants were selected for rail prototrophy on SD medium (0,67% YNB, 2% glucose and required auxotrophy at the concentration of 10 *μ*g ml^-1^).

To culture the auxotrophic parental BY4741 [PIR4-IL1*β*] and the Δ*yca1 *mutant in bioreactor, as well as for inocula development, a defined mineral medium, prepared according to Verduyn *et al*. [30] was employed, containing vitamins and trace elements and made selective without uracil.

For the batch phases of all the experiments, the defined medium was supplemented with casamino acids (BD Bacto™ Casamino Acids, BectonDickinson & Co., Sparks, MD 21152 USA) as the only auxotrophy-complementing amino acid (ACA) source (SDMB medium), the amount of which was determined on the basis of its amino acidic composition (BectonDickinson, technical handbook), and the recommendations on the nutritional requests obtained from the literature [22,23]. SDMB, containing 2% w/v glucose as carbon source, was employed for the batch phase.

For the fed-batch phases of all the experiments, both the feeding solutions employed (called FC and FAC, see below) contained 50% w/v glucose, as well as salts, trace elements, glutammic acid, vitamins, and ACA source. Final salt concentrations per litre were: KH_2_PO_4 _15.70 g, KCl 5 g, MgSO_4 _7H_2_O, 5.83 g, CaCl_2_.2H_2_O, 1.20 g, NaCl 0.44 g, FeSO_4_.7H_2_O 250 mg. Final trace concentrations per litre were: ZnSO_4_.7H_2_O 50 mg, CoCl_2_.6H_2_O 2 mg, CuSO_4_.5H_2_O 40 mg, MnCl_2_.4H_2_O 50 mg and final glutammic acid concentration per litre was 1 g. Final vitamin concentrations per litre were: biotin 4 mg, calcium-pantothenate 40 mg, nicotinammide 90 mg, myo-inositol 50 mg, thiamine HCl 100 mg, pyridoxine HCl 20 mg.

As regards the ACA concentration, two feeding solutions, called FC (feeding with casamino acids) and FAC (feeding with individual amino acids plus casamino acids), were employed. FC contained as ACA source only casamino acids, the amount of which was determined on the basis of its amino acidic composition, the assumed final biomass to be achieved in the bioreactor (60 g l^-1^), and, as above mentioned, the recommendations obtained from the literature [22,23], so that the casamino acid concentration was 50 g l^-1^. In the case of FAC, the final ACA concentration was achieved using both casamino acids, up to 50 g l^-1 ^and the essential individual amino acids (histidine, leucin, and methionine). The overall concentration of each auxotrophy-complementing amino acid in the reservoir was calculated from the equation developed for the time dependent feed flow rate *F(t) *needed to achieve the constant *μ *value imposed, taking into account the value of biomass yield for the given amino acid in aerobic conditions and considering an excess factor of ca. 25% [24].

Development of the inoculum for the fed-batch reactor was made starting from the frozen culture (stored at -80°C in 12.5% v/v glycerol) and ensuring propagation in an Erlenmeyer flask (pre-culture) in the same medium used for the bioreactor.

### Fed-batch cultures

Fed-batch cultures were performed in a 2 l working volume stirred Bioflo 110 (New Brunswick Sc.). The bioreactor was inoculated with an adequate aliquot of a 18 h pre-culture, to give an initial O.D._590 _of 0.200. The SDMB medium was employed for the batch phase; the feeding was represented by either FC or FAC solution described in the previous section.

Fed-batch cultures were performed in three steps. First, the reactor was allowed to proceed in a batch mode (1 l volume) overnight. The second step, (exponential feed phase) started when the glucose of batch phase was exhausted (15-16 h). Then, an exponentially increasing feed was applied, so that the biomass could increase at a constant specific growth rate (*μ*) of 0.16 h^-1 ^selected below the critical value (that is 60% of the *μ*_max _value, the latter being 0.39 h^-1^), to avoid sugar overflow metabolism [26]. Then, the exponential feed was switched to a constant feed phase.

During batch phase, oxygen was supplied by sparging the bioreactor with air at a flow of 1 vvm and the cascade system acted with the agitation speed automatically increasing or decreasing until the DOT set-point (30% air saturation) was reached. The culture pH was maintained at 5.00 by automatic addition of 2 N KOH. During exponential and constant feeding phases, aeration was accomplished as described above, except for the air flow which was of 1.5 vvm and pH maintained by automatic addition of 10% v/v NH_4_OH. The foam level in the bioreactor was controlled by the automatic addition of the antifoam Dow Corning 1510 (dil. 1:10).

All the fed-batch runs were carried out in duplicate.

### Biomass determination and cell viability

Biomass was determined by optical density measurements at 590 nm (O.D_590_) and dry weight determination. Unless otherwise stated, mg of biomass are always referred as dry weight. The calibration curve relating O.D_590 _values to biomass density provided a correlation factor of 2.45 O.D_590 _per mg ml^-1^.

During fed-batch runs, cell viability was determined by viable count on SDMB agar plates, incubated at 30°C for 48 h. *k*_*d *_(the specific death rate constant) was evaluated by plotting the ratio CFU ml^-1^/OD_590 _*vs*. time, where CFU corresponded to the colony forming units on the plates originated from viable cells, and OD_590 _corresponded to the total amount of cells (viable and unviable) in the medium.

The viable cell density *(X*_*V*_) (mg ml^-1^) of each sample collected from the reactor was determined according to the following expression:(1)

Where *X*_*V*0_, the biomass density at the beginning of a fed-batch run was spectrophotometrically determined, as mentioned above. It was assumed that, at the beginning of fed-batch phase (t_0 _= 0), all the yeast cells were viable.

### Analyses

Samples were quickly withdrawn from fed-batch cultures, filtered on 0.45 *μ*m GF/A filters (Millipore, Bedford, MA USA) and filtrates analysed to determine residual glucose, ethanol and interleukin-1*β *concentrations.

Residual glucose (mg ml^-1^) in the medium was determined by GOD-Perid from R-Biopharm (Roche, Mannheim, Germany) or a method for reducing sugars [31], the latter was employed when high ethanol concentration in the medium caused enzyme denaturation of the GOD-Perid kit. Ethanol production was measured with the enzymatic kit from R-Biopharm (Roche, Mannheim, Germany).

Interleukin-1*β *was determined in quadruplicate by immuno-blot analysis in Bio-Dot^® ^Microfiltration Apparatus (Bio-Rad, Hercules, CA, USA), as already reported [18]. All samples were analysed in triplicate and the values of standard deviation obtained varied between 1 and 2%.

### Mathematical model

The mathematical model needed to describe the fed-batch cultures carried out with the parental *S. cerevisiae *BY4741 and the Δ*yca1 *mutant was developed on the basis of balances of components of interest: biomass, growth limiting substrate (glucose) and product (IL-1*β*).

To describe the change with time of the arbitrary component with concentration *y *in the reactor, the following simplified general mass balance equation was used:(2)

taking into account that, for a fed-batch reactor, the total mass balance on the reaction volume is *dV/dt *= *F*.

The solution of the differential equation obtained for each component of the culture system (Table 1) was numerically found starting from given initial values, using the Eulero method. To assign the parameter values (Table 2), simulations were compared to the experimental data, so as to find a parameter set which gave the best fit of the model to the experimental data. The evaluation of the fit of the model to the experimental data was done by minimising the sum of squared errors between the model and the experimental data.

The exponential profile of flow rate *F(t) *was obtained from the mass balance on limiting substrate and calculated according to Enfors [26] throughout the assumption of a quasi-steady state on the glucose balance, made with the scope to maintain glucose concentration in the feeding at levels sufficient to promote a fully respiratory metabolism.

The mathematical model was developed considering only the viable biomass, the density of which, *Xv *(g l^-1^), was calculated according to equation (1). It was considered that the specific growth rate (*μ*) imposed to build up the exponential feeding profile was maintained in the time interval 0 ≤ t< t_1 _and exponentially decreased when t ≥ t_1 _(Table 1). Further, the specific glucose consumption rate was split into the components, q_G _and q_m _(Table 1), which accounted for energy and precursors for growth and energy demand for maintenance, respectively. Product yield (Y_p/s_) remained unvaried along the entire runs (Table 1).

Some other simplifications and assumptions were made, which are listed below:

i) the batch growth phase was not considered in the modeling procedure. It was only employed to develop a suitable inoculum after the initial glucose charge was consumed and to generate the initial experimental conditions *X*(*t*_0_), *V*(*t*_0_) for the following fed-batch phases;

ii) the same correlation factor of 2.45 O.D_590 _per mg ml^-1 ^was employed to determine the viable biomass density, assuming that yeast cell size did not change either within the entire yeast population or with time.

### List of symbols

The list of symbols used for the mathematical model and their definitions is reported below:

*α *= constant for parabolic increase of specific glucose consumption for maintenance in the interval t_1 _≤ t < t_2 _[t^-3^]; *β *= constant for parabolic increase of specific glucose consumption for maintenance in the interval t_1 _≤ t < t_2 _[t^-2^]; *γ *= constant for parabolic increase of specific glucose consumption for maintenance in the interval t_1 _≤ t < t_2 _[t^-1^]; *δ *= constant for exponential decrease of specific growth rate [h^-1^]; *θ *= constant for exponential increase of specific glucose consumption rate for maintenance [h^-1^]; *F *= glucose feed rate at time *t *[ml h^-1^]; *G *= residual glucose concentration [g l^-1^]; *G*_0 _= initial residual glucose concentration [g l^-1^]; *G*_*R *_= glucose concentration in the reservoir [g l^-1^]; *k*_*d *_= specific death rate [h^-1^]; *λ *= constant for exponential increase of specific glucose consumption rate for growth [h^-1^]; *μ*_*i *_= specific growth rate between in the ith interval [h^-1^]; *μ*_1 _= specific growth rate between 0_≤ _t ≤ t_1 _[h^-1^]; *μ*_2 _= specific growth rate for t > t_1 _[h^-1^]; *P *= interleukin-1*β *concentration [g l^-1^]; *P*_0 _= initial interleukin-1*β *concentration [g l^-1^]; *q*_*Gi *_= specific glucose consumption rate for growth in the ith interval [g g^-1 ^h^-1^]; *q*_*G*1 _= specific glucose consumption rate for growth in the interval 0_≤ _t < t_1 _[g g^-1 ^h^-1^]; *q*_*G*2 _= specific glucose consumption rate for growth in the interval t_1 _≤ t < t_2 _[g g^-1 ^h^-1^]; *q*_*mj *_= specific glucose consumption rate for maintenance in the jth interval; *q*_*m*1 _= specific glucose consumption rate for maintenance in the interval 0_≤ _t < t_1 _[g g^-1 ^h^-1^]; *q*_*m*2 _= specific glucose consumption rate for maintenance in the interval t_1 _≤ t < t_2 _[g g^-1 ^h^-1^]; *q*_*m*3 _= specific glucose consumption rate for maintenance in the interval t ≥ t_2 _[g g^-1 ^h^-1^]; *r*_*y *_= volumetric reaction rate of component *y *[g l h^-1^]; *t *= time [h]; t_1 _= time of fed-batch run in correspondence of which the variation of both specific growth rate and glucose consumption start [h^-1^]; *t*_2 _= time in correspondence of which the exponential feeding switched to a constant feeding [h]; *V *= culture volume at time *t *[l]; *V*_0 _= initial culture volume [l]; *X*_*V *_= viable biomass concentration [g l^-1^]; *X*_*V*0 _= initial viable biomass concentration [g l^-1^]; *y *= concentration of variable of interest [g l^-1^]; *y*_*i *_= concentration of variable of interest in the inlet [g l^-1^]; *Y*_*P*/*X *_= IL-1*β *yield from biomass.

## Competing interests

The authors declare that they have no competing interests.

## Authors' contributions

LP performed the experimental part of the work, carried out the analysis and interpretation of the data and developed the mathematical model, EA participated in the engineering of *S*. *cerevisiae *BY4741 and the immunoassays, has been involved in drafting the manuscript for important intellectual content, VP and CM contributed equally to obtain the deletion *yca1 *mutant, JZ supervised the engineering of *S. cerevisiae *BY4741 for heterologous protein expression; PP wrote the manuscript, conceived and supervised the study. All authors have read and approved the final manuscript.
